# An unusual case of spleen metastasis from carcinoma ex pleomorphic adenoma of the parotid gland

**DOI:** 10.1186/1477-7819-12-18

**Published:** 2014-01-23

**Authors:** Luigi Rossi, Erika Giordani, Antonella Fontana, Claudio Di Cristofano, Giuseppe Cavallaro, Oreste Bagni, Luca Filippi, Loredana Bianchi, Giulia Rinaldi, Francesca Perrone Congedi, Anselmo Papa, Davide Caruso, Monica Verrico, Gianfranco Silecchia, Silverio Tomao

**Affiliations:** 1 Department of medico-surgical sciences and biotechnologies, Faculty of Pharmacy and Medicine, “Sapienza” University of Rome, Oncology Unit, ICOT, Via Franco Faggiana, 1668, 04100 Latina, Italy; 2 Department of Radiotherapy, Santa Maria Goretti Hospital, Via Guido Reni, 3, 04100 Latina, Italy; 3 Department of Medico-Surgical Sciences and Biotechnologies, Faculty of Pharmacy and Medicine, “Sapienza” University of Rome, Pathology Unit, ICOT, Via Franco Faggiana, 1668, 04100 Latina, Italy; 4 Department of medico-surgical sciences and biotechnologies, Faculty of Pharmacy and Medicine, “Sapienza” University of Rome, General Surgery Unit, ICOT, Via Franco Faggiana, 1668, 04100 Latina, Italy; 5 Department of Nuclear Medicine, Santa Maria Goretti Hospital, Via Guido Reni, 3, 04100 Latina, Italy

**Keywords:** Carcinoma ex pleomorphic adenoma, Parotid tumours, Pleomorphic adenoma, Splenic metastases

## Abstract

Carcinoma ex pleomorphic adenoma is a rare tumor arising from the salivary glands that spreads through direct extension, through the lymphatic vessels, and, rarely, hematogenously. When distant metastases have been found, they have been reported mainly in the lung. We present an unusual case of carcinoma ex pleomorphic adenoma of the parotid gland with splenic metastases. The patient presented with a primary carcinoma ex pleomorphic adenoma of the parotid gland and he underwent a total parotidectomy with laterocervical lymphadenectomy ipsilateral and adjuvant radiation therapy to the right parotid area. One year later, the patient showed an ipsilateral supraclavicular lymph node recurrence, treated with surgery and radiation therapy. Two more years later, the patient developed lung and splenic lesions, detected through CT and PET. He underwent splenectomy and pathologic assessment of the specimen showed metastatic carcinoma ex pleomorphic adenoma. To our knowledge, there is no reported case of a carcinoma ex pleomorphic adenoma metastasizing to the spleen. Patients treated for carcinoma ex pleomorphic adenoma should be investigated for distant metastases with a long-term follow-up examination for local and distant metastases and new splenic lesions in these patients should be investigated.

## Background

Pleomorphic adenoma (PA) is the most common tumor of the salivary glands (it includes about 65% of all salivary gland tumors). PA originates more commonly in parotid glands (65% to 75%), 8% in submandibular glands, and only 6% to 7% in minor salivary glands. Generally, it is considered a benign and slow-growing tumor, but about 1.6% to 7.5% of PA shows malignant changes during its natural history, including three different types: carcinoma ex pleomorphic adenoma (CXPA), also called malignant mixed tumor, carcinosarcoma, and metastasizing PA (MPA); the latter two are exceedingly rare [1,2].

Histologically, MPA cannot be differentiated from a benign PA (it consists of the presence of both epithelial and mesenchymal benign elements), but it is a malignant aggressive entity; in fact, during its natural history it manifests local or distant metastasis [3]. Carcinosarcoma is composed of malignant epithelial and mesenchymal components and it has a very aggressive course. CXPA represents approximately 3% to 5% of all salivary gland neoplasms and it predominantly arises from major salivary glands, more commonly from the parotid gland [4]. Misdiagnosis is common because the residual PA component may be small, and because various carcinoma subtypes may be present [5].

CXPA is a rare aggressive epithelial malignancy with poor prognosis, frequently leading to local metastasis and with a high mortality. Gnepp et al. found that 5-year survival ranged from 25% to 65% and that the detection of metastases is considered pre-terminal [6]. Important prognostic factors of CXPA in the major salivary glands are stage, lymph node involvement, tumor type, histological grade, perineural invasion, and extent of invasion [7]. However, because of its low incidence, no standard treatment has been described so far. Surgery is the primary treatment for CXPA, and postoperative radiation therapy plays an important role. Presently, no evidence suggests that adjuvant chemotherapy can improve CXPA prognosis [8]. CXPA spreads through a direct locoregional extension or lymphatic spread (typically to the cervical lymph nodes), and rarely hematogenously. When distant metastases are found, they have been reported mainly in the lung, bone, and liver [9].

The clinical case reported below refers to the unusual experience of our patient, affected by a CXPA metastatic to the spleen. As far as we know, this is the first case to be reported in the literature.

## Case presentation

A 66-year-old man, presented to our hospital in February of 2010, about five months after the occurrence of soft tissue swelling on the right laterocervical area that had gradually increased in size. An ultrasound and a CT scan of the neck showed an inhomogeneous and irregular lesion in the right laterocervical area. A biopsy of this lesion was performed, indicating a carcinoma ex pleomorphic adenoma of the right parotid gland.

The patient underwent adjuvant radiation therapy consisting of 1.50 Gy in 25 fractions and a boost of 2.10 Gy in 5 fractions to the right parotid area.

**Figure 1:**
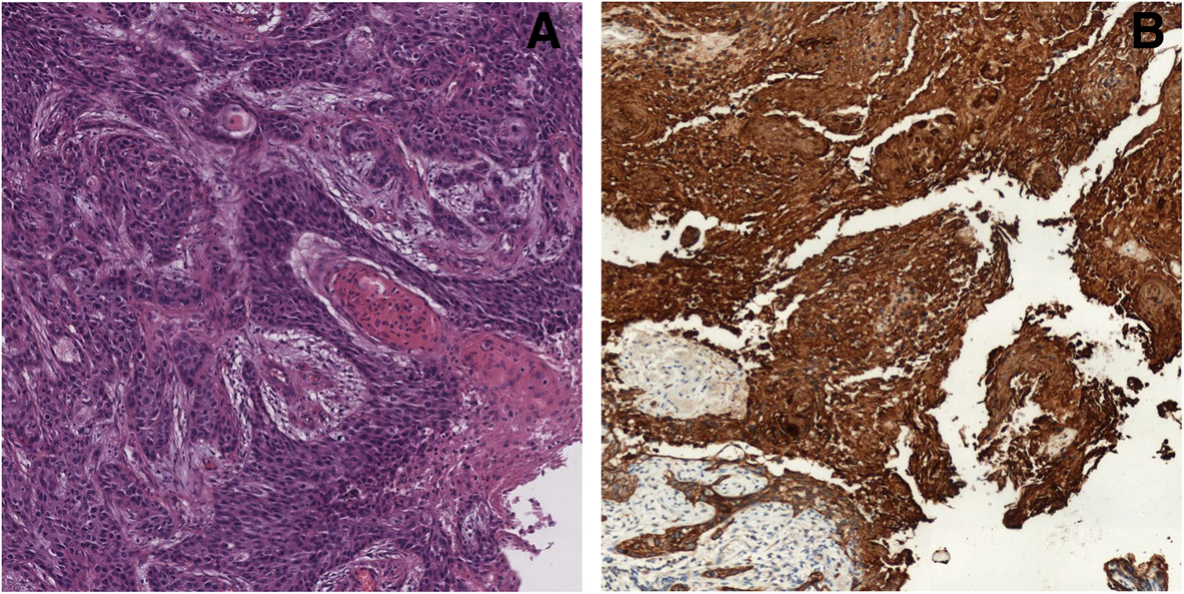
**Histologic specimen of carcinoma ex pleomorphic adenoma of the parotid.** Image shows the poorly differentiated malignant epithelial component in pleomorphic adenoma **(A)**. Immunohistochemical staining demonstrated strong and diffuse positivity for a cocktail of cytokeratins **(B)**.

After a staging CT scan and a total parotidectomy with ipsilateral laterocervical lymphadenectomy, a CXPA of the right parotid infiltrating the skin surface and the next muscle tissue of stage pT4a pN0 M0 was confirmed. Histologic examination showed a poorly differentiated malignant epithelial component originated from a pleomorphic adenoma. Immunohistochemically, cells were positive for p63 and a cocktail of cytokeratins. Moreover, Ki67 labeling index was 50% (Figure 1).

The patient underwent adjuvant radiation therapy consisting of 1.50 Gy in 25 fractions and a boost of 2.10 Gy in 5 fractions to the right parotid area.

In March of 2011, during a surveillance CT of the neck an irregular and inhomogeneous lesion measuring 4 cm in the right supraclavicular region was detected. The patient underwent dissection of right supraclavicular lymph nodes with histopathological confirmation of a secondary localization of CXPA of salivary glands. The patient was subjected to radiation therapy consisting of 50 Gy in 25 fractions to the right supraclavicular region.

**Figure 2:**
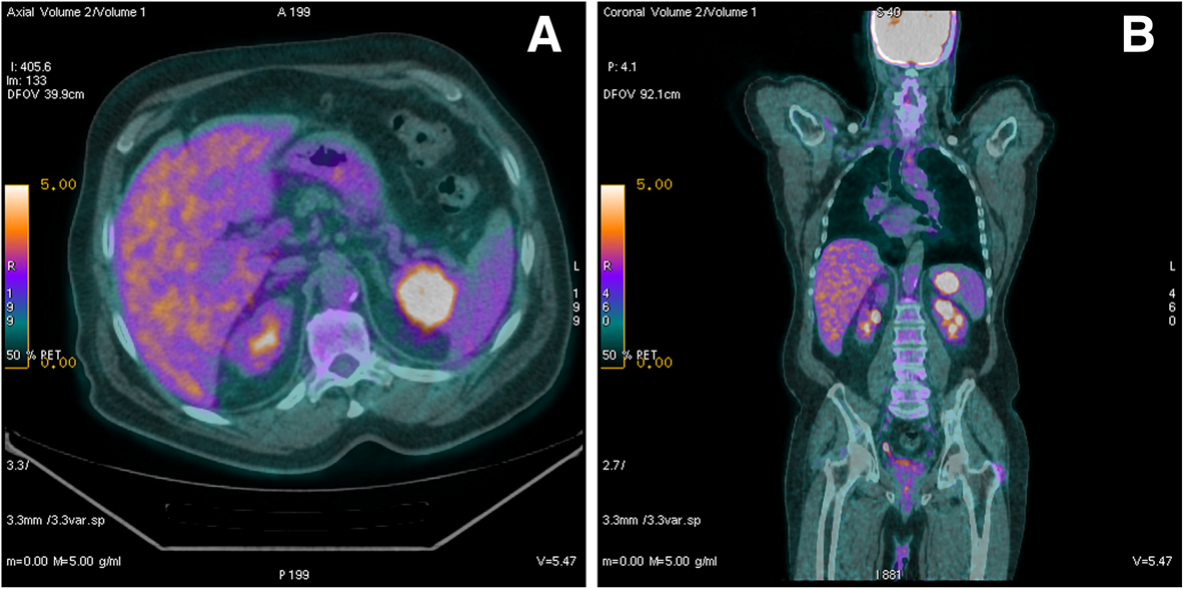
^**18**^**F-FDG PET images of splenic metastasis.** Axial section **(A)** and coronal section **(B)**.

Follow-up of the patient demonstrated no locoregional recurrence or distant metastases; however, in February of 2013 the patient underwent a surveillance CT scan showing a low-density nodular lesion measuring 1.2 cm in the apical segment of the upper lobe of the left lung and another similar lesion measuring 2.5 cm in the superior-medial portion of the spleen. Indeed, a PET scan confirmed the presence of two hypercaptant lesions in the left lung (SUV 4.1) and in the spleen (SUV 10.6) (Figure 2).

The patient was referred to the Hematology Department, who performed a bone marrow biopsy and ruled out a hematological disease.

**Figure 3:**
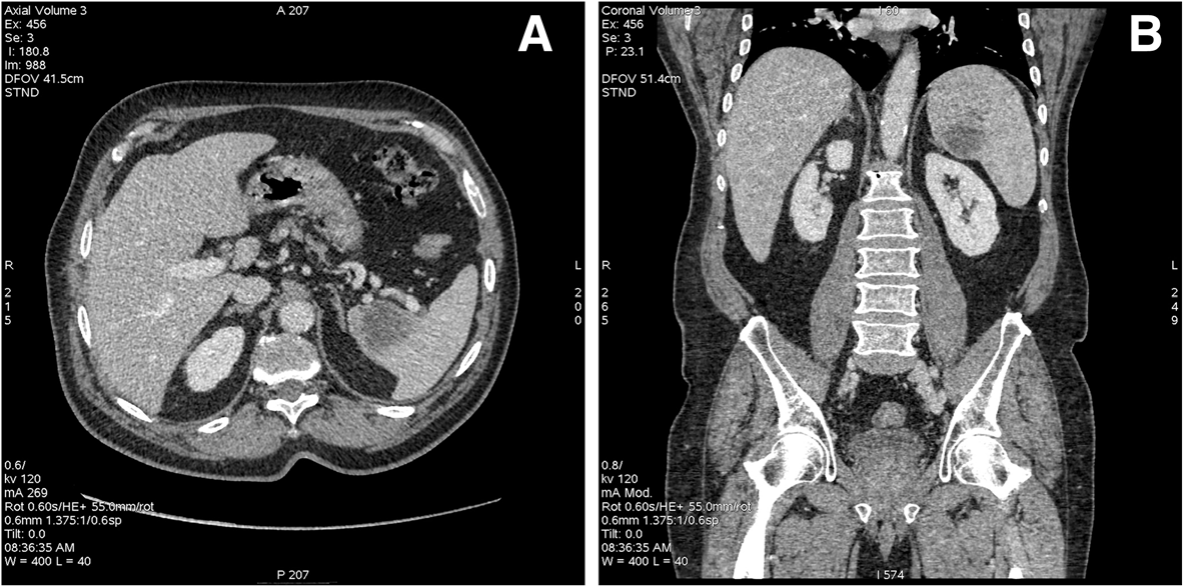
**CT images of splenic metastasis.** Axial section **(A)** and coronal section **(B)**.

In April of 2013, the patient underwent a new CT scan showing a low-density nodular lesion measuring 1.7 cm in the apical segment of the upper lobe of the left lung that had increased in volume and a hypovascular lesion measuring 4.8 cm in the superior-medial portion of the spleen, which had also increased in volume (Figure 3). The patient was referred to the General Surgery Department and underwent a laparoscopic splenectomy. This operation became hard due to the presence of metastasis displacing the hilar vessels. Histologic examination revealed that three out of four of the removed lymph nodes as well as the spleen were involved by atypical epitheliomorphic cells, arranged in solid nests and cords with a round or oval nucleus and a large eosinophilic cytoplasm. Few cellular monstrosities were present, while necrosis was not observed. Immunohistochemically, these CXPA cells were specifically positive for cytokeratin 7, S-100 protein, and p63, but were negative for cytokeratin 20, smooth muscle actin, and gross cystic disease fluid protein-15 (GCDFP-15). On the other hand, Ki67 labeling index was 50%.

**Figure 4:**
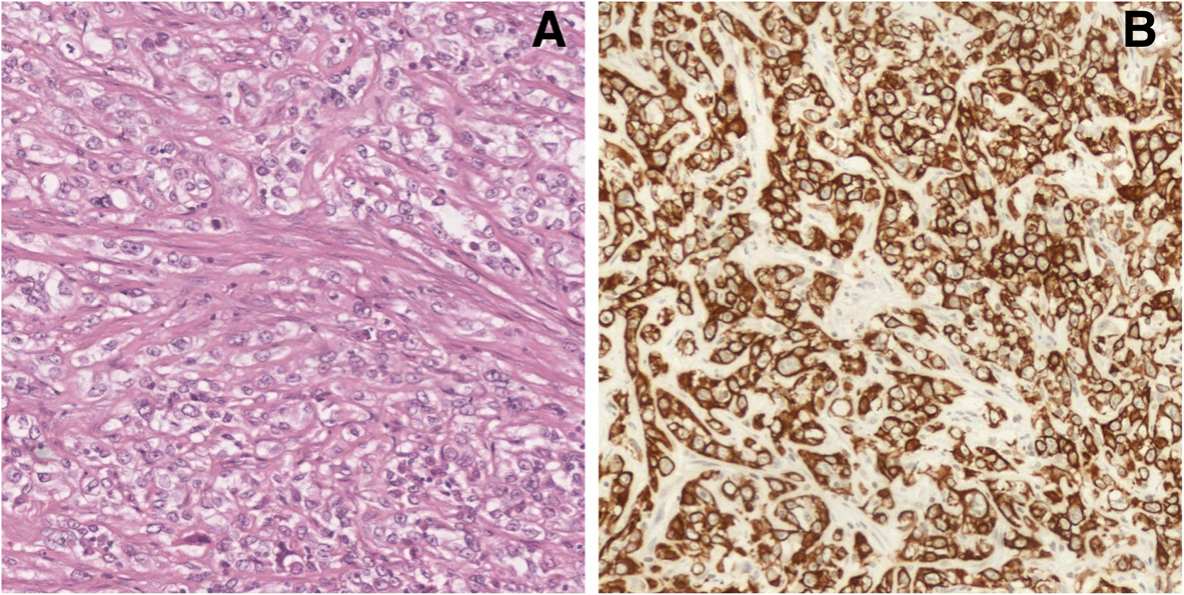
**Histologic specimen of splenic metastasis.** The spleen is involved by atypical epitheliomorphic cells, arranged in solid nests and cords with a round or oval nucleus and a large eosinophilic cytoplasm **(A)**; immunohistochemical staining demonstrated positivity for cytokeratin 7 **(B)**.

Morphology and immunohistochemical panels indicated splenic and lymph node localization of a poorly differentiated epithelial neoplasm, which was difficult to classify histopathologically, probably originating from the salivary glands (Figure 4).

## Conclusion

PA of the salivary glands is generally considered a benign neoplasm, but in 2% to 9% of cases it can undergo to carcinomatous transformation (i.e., CXPA) [10]. CXPA is a rare and poorly understood neoplasm developing from either a long-standing primary or recurrent pleomorphic adenoma. It typically occurs in patients in the 6^th^ to 8^th^ decade of life. Increased preoperative duration of a PA increases the risk of malignant transformation into CXPA [11]. Our patient was a 66-year-old man who underwent surgery five months after the occurrence of soft tissue swelling on the right laterocervical area.

CXPA contains both benign PA and carcinomatous components in different proportions. In some cases, the malignant component can completely replace the mixed tumor, which usually leads to misdiagnosis. As reported herein, it is worth noting that only the carcinomatous component was present in the metastatic lesion.

The mechanism of malignant transformation of PA is still unclear, although it probably depends on accumulation of genetic instabilities [12]. CXPA is sub-classified into non-invasive, minimally invasive (about 1.5-mm penetration of the malignant component into the extra capsular tissue), and invasive (more than 1.5-mm penetration into the extra capsular tissue); treatment strategies usually depend on this sub-classification [2].

Although any form of the above forms of carcinoma can be observed as well as a mixture of subtypes, the WHO states that the components of PA are most frequently a poorly differentiated carcinoma (e.g., salivary duct carcinoma or adenocarcinoma, not otherwise specified) or an undifferentiated carcinoma [2]. Advanced T stage and lymph node involvement have been identified as significant prognostic factors for an unfavorable clinical outcome [7]. The extent of tumor infiltration beyond the capsule and, therefore, extraparotid invasion have been found to correlate with CXPA recurrence and survival and they are some of the most reliable prognostic factors [7].

Vascular and perineural invasion has a statistically significant effect on distant metastases, tumor-specific survival, and overall survival, while high-grade tumors are more likely to predict unfavorable clinical outcomes [4,7]. The presence of myoepithelial carcinoma subtype appears to increase the risk of recurrence and p63 antigen may be an useful marker of myoepithelial cells in salivary gland neoplasms [13]. Aggressive tumors have a high MIB1 index (Ki67), which results in rapid growth [14].

Herein, the negative prognostic factors that indicated an aggressive tumor biology as evidenced by its unusual spread to the spleen were the advanced T stage (pT4a) and the invasiveness. In fact, the primary tumor infiltrated the skin surface and the next muscle tissue, while no vascular or perineural invasion was described. Initially, laterocervical lymph nodes were not involved, but 1 year later the tumor relapsed to the supraclavicular lymph nodes. Furthermore, histologic examination of the primary and secondary lesions revealed a poorly differentiated epithelial neoplasm that was immunohistochemically positive for p63. Moreover, Ki67 labeling index was high (50%).

Obviously, the prognosis also depends on the completeness of the tumor resection and on the presence of locoregional recurrence or distant metastases. The prognosis after detection of progression or recurrence is poor, with a median survival of less than 1 year. [7]CXPA can be asymptomatic and often has similar clinical presentations as PA; however, patients have a poor prognosis due to infiltrative and destructive behavior and thus, early and accurate diagnosis and aggressive surgical treatment can increase their survival rates [4]. Total or radical parotidectomy is indicated for frankly invasive CXPA and neck dissection should be performed for the majority of CXPA patients, except for some intra-capsular or minimally invasive diseases [4]. Postoperative radiation therapy is often used as an adjuvant therapy in patients with adverse risk factors and it significantly improves local control, but it does not translate into a survival advantage [4,7].

In the evaluation of salivary gland tumors, the use of 18-fluoro-2-deoxy-D-glucose positron emission tomography (^18^F-FDG PET) remains a matter of debate. Kim et al. illustrated an association between high FDG uptake and Glut-1 overexpression in CXPA, and they noticed that it could offer a basis for the clinical application of ^18^F-FDG PET and Glut-1 for differential diagnosis between CXPA and PA [15]. Otsuka et al. showed that FDG PET had a significant impact on the management of patients with salivary malignant tumors in both the initial staging and restaging [16]. In our case, despite the proper therapeutic management, the patient relapsed 1 year later. Moreover, ^18^F-FDG PET was useful for detection of metastases.

Generally, the spleen is a rare site of metastases. The most common neoplasms spreading to the spleen reported in the literature are breast, lung, colon and rectum, ovary, and stomach. Splenic metastases arising from head and neck cancers are exceedingly rare. Lam et al. reviewed autopsies over a 25-year period and found only 5 cases of splenic metastases originating from the nasopharynx and 1 from the larynx [17]. There is 1 case report of squamous cell carcinoma of the tonsil metastatic to the spleen presenting as a splenic rupture [18], while Raval et al. described a case of squamous cell carcinoma of the neck with splenic metastases treated with laparoscopic splenectomy [19].

CXPA usually spreads through a direct locoregional extension and local lymphatic spread is the most common means by which these tumors metastasize (typically to the cervical lymph nodes). While reports of local recurrence of this cancer are numerous, few cases of distant hematogeneous metastases have been reported. Distant metastases have been reported to occur in as many as 44% of patients with CXPA [9]. Previously reported sites of hematogeneous metastases include the lungs (the most common site), pleura, pharynx, kidney, ocular choroid, liver, bone, brain, and spinal cord [20-24].

In our case, both regional lymphatic and distant hematogeneous spread were identified. This case is exceptional because, to our knowledge, it is the first case of salivary gland tumor metastatic to the spleen and the first case of CXPA metastatic to the spleen.

In conclusion, when recurrence or distant metastases occur in CXPA, survival is very low. Although distant metastases are rare, our case report remarks that patients treated for CXPA should be investigated for distant metastases with a long-term follow-up examination for local and distant metastases. Moreover, our case underlines that salivary gland tumors (even CXPA) can also metastasize to the spleen and lesions seen in the spleen in a patient with salivary gland tumors (even CXPA) should be further investigated with additional imaging.

## Consent

The patient has given his consent for the publication of this case report and any accompanying images.

## Abbreviations

CXPA: Carcinoma ex pleomorphic adenoma; MPA: Metastasizing PA; PA: Pleomorphic adenoma.

## Competing interests

The authors declare that they have no competing interests.

## Authors’ contributions

TS was the coordinator of the paper; RL and GE was the main authors of the manuscript; FA SG and GC corrected the language form; DCC, BL and RG elaborated histological images, BO and FL elaborated Radiological images, PA and PGF performed a literature review, VM and CD collected and studied the bibliography. All authors read and approved the final manuscript.
